# Chronic illness and multimorbidity among problem drug users: a comparative cross sectional pilot study in primary care

**DOI:** 10.1186/1471-2296-10-25

**Published:** 2009-04-21

**Authors:** Walter Cullen, Sarah O'Brien, Austin O'Carroll, Fergus D O'Kelly, Gerard Bury

**Affiliations:** 1 UCD School of Medicine and Medical Science, Coombe Healthcare Centre, Dublin, Ireland; 2 Mountjoy Street Family Practice, Dublin, Ireland; 3 Department of Public Health and Primary Care, Trinity Centre for Health Sciences, Dublin, Ireland; 4 Rialto Medical Centre, Dublin, Ireland

## Abstract

**Background:**

Although multimorbidity has important implications for patient care in general practice, limited research has examined chronic illness and health service utilisation among problem drug users. This study aimed to determine chronic illness prevalence and health service utilisation among problem drug users attending primary care for methadone treatment, to compare these rates with matched 'controls' and to develop and pilot test a valid study instrument.

**Methods:**

A cross-sectional study of patients attending three large urban general practices in Dublin, Ireland for methadone treatment was conducted, and this sample was compared with a control group matched by practice, age, gender and General Medical Services (GMS) status.

**Results:**

Data were collected on 114 patients. Fifty-seven patients were on methadone treatment, of whom 52(91%) had at least one chronic illness (other then substance use) and 39(68%) were prescribed at least one regular medication. Frequent utilisation of primary care services and secondary care services in the previous six months was observed among patients on methadone treatment and controls, although the former had significantly higher chronic illness prevalence and primary care contact rates. The study instrument facilitated data collection that was feasible and with minimal inter-observer variation.

**Conclusion:**

Multimorbidity is common among problem drug users attending general practice for methadone treatment. Primary care may therefore have an important role in primary and secondary prevention of chronic illnesses among this population. This study offers a feasible study instrument for further work on this issue. (238 words)

## Background

Injecting drug users have an increased mortality compared to the general population. Mortality rates of 13–30 per thousand persons per year (an age-adjusted mortality rate of 6.9–22.0) have been reported among injecting drug users, with opiate overdose and HIV infection the major causes of death [1-5].

The introduction of 'harm reduction' interventions (ie interventions designed to prevent problems associated with problem drug use, eg oral methadone treatment, needle and injecting paraphernalia exchange programmes) has, in addition to advances in HIV treatment, been an important factor in reducing mortality/increasing life expectancy among problem drug users [6-8]. One might therefore expect an 'epidemiological shift', as chronic illnesses and their complications replace infectious diseases and drug overdose as causes of death. In this respect, an association between problem drug use and subsequent decrease in general self-rated health has been demonstrated [9].

While bloodborne virus infections (in particular hepatitis C [10-12]), psychiatric illness [13] and to a lesser extent, problem alcohol use [14] are all recognised as chronic medical problems that are more prevalent among injecting drug users, our understanding of other 'common' chronic illnesses among injecting drug users is less advanced. While much is known about the epidemiology and management of diabetes, cardiovascular disease and chronic respiratory illness (eg asthma, COPD) and while the prevention and management of these chronic illnesses has been identified as a priority issue for population health in Ireland and globally [15,16], no data have reported on the epidemiology or care of these illnesses among current or former drug users.

The aims of this study were:

- to determine chronic illness/multimorbidity prevalence and health service utilisation among problem drug users attending primary care for methadone treatment and to compare these rates with matched 'controls';

- to develop and pilot test a valid study instrument.

## Methods

### Setting

The study was carried out in Dublin city, where an estimated 16.0 per thousand of the population currently use illicit opiates [17]. In Ireland, addiction treatment is currently provided by specialist addiction treatment services, including a central Drug Treatment Centre Board, regional addiction centres, community-based projects (satellite clinics) and by primary care. Most recent data reported that 7845 patients were treated for problem drug use by these agencies in 2002, with opiates the most common drug for which people attended for treatment (86% of total) [18].

In recent years, the number of general practitioners (GPs) prescribing methadone has increased in Ireland, the UK and elsewhere in the EU [19-23]. To prescribe methadone in Ireland, GPs must complete special training and are subject to clinical audit, with GPs who provide methadone treatment for 15 or more patients subject to more regular audit and advanced training ('level 2' GPs) [24]. This system is analogous to the 'GPs with a special interest' ('GPWsi') model currently operating in the UK [25]. Initiation of methadone treatment is only permitted in specialist addiction clinics or by 'level 2' GPs [24]. In all other cases, chaotic opiate users are initially cared for in a specialist addiction clinic and their care then transferred to general practice when stable. In circumstances where illicit drug use of patients attending general practice becomes 'chaotic', addiction care is transferred to the specialist addiction services.

The study was conducted in three large group general practices (see Table 1), all situated in areas of high deprivation and with practice populations predominantly eligible for General Medical Services (GMS) scheme (the scheme that at the time of the study provided free primary care services to approximately 30% of the Irish population on the basis of age over 70 years or low income). Each practice used both electronic and hard copy clinical records, but at the time of the study, each had been using electronic records alone for all new clinical and prescribing notes for at least six-months. Each practice was research-active and formally affiliated with one of Dublin's three medical schools.

**Table 1:** Characteristics of practices that participated in study.

Practice	Description	Approximate number of patients on methadone treatment at practice	Clinical records
A	3.0 FTE doctor teaching general practice in Dublin's south inner city	19	Combined electronic/paper
B	Four doctor teaching general practice in Dublin's south inner city	45	Combined electronic/paper

C	2.5 FTE general practice in Dublin's north inner city	70	Combined electronic/paper

### Subjects

All patients attending each practice for methadone treatment on 1^st ^July 2008 were eligible for the study. A considerable variability in numbers of patients attending the three practices for methadone existed at the time of the study, with 19 the smallest number of patients attending one of the practices for methadone treatment and hence 19 cases were sampled at each practice.

#### Sampling of 'cases'

At each participating practice, the researcher and GP identified cases for inclusion in the study by random number sampling from a numbered list of patients attending that practice for methadone treatment (in Ireland, GPs are not allowed to issue a prescription for methadone for any more than two weeks and are remunerated on a 'per consultation' basis).

#### Sampling of 'controls'

Controls were matched for practice, age, GMS scheme membership and gender. Potential controls were identified by running a query of 'active patients' registered on each practice's electronic practice management information system using the matching criteria. This list of potential controls was then numbered and one control identified using random number sampling.

### Data collection and validation

Data was collected from clinical records. A study instrument was developed for the purpose of this study (see Appendix 1). The instrument was based on another instrument routinely used to collect health information on current or former injecting drug users in Ireland, the Health Research Board National Drug Treatment Reporting System (NDTRS) [26]. Data were therefore collected on:

• Demography

• Addiction care

• Chronic illnesses

• Presentations with acute illnesses

• Prescribing

• Health service utilisation (including: attendance at primary care, secondary care, out of hours services, emergency departments and diagnostic investigations).

Data were collected by four researchers over the course of two weeks in July 2008: one researcher (SOB) collected data on all records and at each practice, one of the GPs at that practice (WC, FDOK or AOC) also collected data. Data was extracted from the 'diagnosis' 'prescribing', 'consultation', and 'communication' modules from individual electronic clinical records. In addition, paper records were reviewed for: 'active diagnoses/chronic illnesses', and hospital referral/update/discharge letters.

At consecutive intervals, the researchers compared the data collected to determine the degree of inter-observer variability; this check was conducted after six, 12, 19, 38 and 57 records of patients on methadone had been reviewed. At each review, questions with an inter-observer agreement of less than 90% and questions where data extraction had proved problematic were revised and these revisions were incorporated into subsequent iterations of the study instrument. A log of the data collection process was maintained, with the amount of data collected in one three-hour sessional block and problems in data collection recorded.

### Data analysis

Data were analysed using Statistical Packages for the Social Sciences (SPSS) version 12.0. Analytical techniques included Pearson's chi squared test to determine the significance of associations between categorical variables. Odds ratios and their 95% confidence intervals (CI) were used to compare rates of chronic and acute illness and health service utilisation between the two groups (cases and controls).

### Ethical considerations

All data were collected and/or reviewed by a GP with clinical responsibility for the patient's ongoing care. The strategy whereby GPs were involved in data collection was adopted for two reasons: to optimise data accuracy and in case any health issue was identified that may require a subsequent clinical intervention. Any subsequent clinical interventions were not included in the data collection.

The Irish College of General Practitioners Research Ethics Committee approved this study. We did not seek patient consent for review of clinical records by the researcher/their GP. However, ethical safeguards included the following:

- all data were collected anonymously and any details that could potentially identify individuals were removed after data collection;

- all members of the research team were nominated as 'agents' of each practice and each signed a confidentiality agreement with the other participating practices.

## Results

### Feasibility and validity of data collection

Data were collected on a sample of 114 patients attending three general practices: 57 'cases' attending for methadone treatment (19 per practice) and 57 'controls' (19 per practice) matched by practice, age, gender and GMS status. While considerable inter-observer variation was apparent at the initial stages of data collection, this variation diminished as the study instrument was subsequently modified (See Table 2). For the final seven cases at Practice A, the two researchers disagreed on the answers to 2/36 questions. In addition, later versions of the study instrument facilitated a more efficient data collection process that allowed one researcher to collect data on 19 patients in three three-hour 'sessions' in the case of Practice B and two three-hour 'sessions' in the case of Practice C.

**Table 2:** Description of data collection process from clinical records of patients being prescribed methadone.

Practice	Clinical records	Items with inter-observer disagreement/total	Workload (in number of three-hour sessions) involved in data collection to collect data	
			
			Lead researcher	GP researcher
			2.0	1.0
	7–12	7/34	1.5	1.0
	13–19	2/36	1.0	0.5
B	20–38	0/39	3.0	0.5

C	39–57	0/39	2.0	0.3

### Demographic and addiction characteristics of patients on methadone treatment

The mean age of 'cases' was 37.2 years (standard deviation: 7.25 years), 42(74%) were male, 41(72%) had GMS cover, all were Irish nationals and 56(98%) were documented as living in stable accommodation.

Considerable lifetime contact with the practice was observed: 16 patients (28%) had been attending the practice for less than 5 years, 21 patients (37%) for 5–10 years and 20 patients (35%) for in excess of 10 years. Treatment of illicit drug use (20 patients), registration for general medical care (20 patients) and being referred by specialist addiction treatment services for methadone treatment (17 patients) were the reasons why patients had first attended (see Table 3). All patients that had been referred for methadone treatment by the specialist addiction treatment services had been with the practice for 10 years or less.

**Table 3:** Reasons why, and time since, patients on methadone first attended practice.

Reason first attended practice	First attended the practice <10 years ago	First attended the practice >10 years ago	Total
Treatment of illicit drug use	11	9	20
Referred by addiction services for methadone treatment	17	0	17
General medical care	9	11	20

Total	37	20	57

Considerable contact with specialist addiction services (both lifetime and since commencing methadone treatment at the practice) was also observed among the sample. All patients had been prescribed methadone by specialist addiction services prior to attending the general practice for methadone treatment and 14(25%) patients had been referred back to specialist addiction services for methadone treatment since commencing methadone treatment at the practice (six in the previous year).

The mean daily dose of methadone prescribed (on the last issued prescription) was 66 milligrammes (standard deviation: 26 milligrammes). Tobacco/cigarette smoking status was recorded in the records of 39 patients (68% of total), of whom 37 were recorded as smokers.

### Morbidity among patients on methadone treatment

Fifty-two patients (91%) had a chronic illness (in addition to substance/opiate/drug use) documented in their clinical record (mean chronic illnesses per patient: 2.6, standard deviation: 1.7). Table 4 indicates the number of patients with pre-determined chronic illnesses that were sought during the data collection and in the case of each, the number who had attended secondary care for that problem. Hepatitis C, depression, asthma and HIV/AIDS were the most common illnesses (38, 20, 14 and 8 patients respectively). A wide range of chronic illnesses that had not been pre-determined were also recorded among patients on methadone treatment. Of these 47 chronic illnesses, back pain (5 patients), gastritis/chronic dyspepsia/gastro-oesophageal reflux (4 patients) and DVT/varicose veins/thrombophlebitis (four patients) were the most common. Thirty-nine patients (68% of total) were on regular prescribed medication, in addition to methadone, (mean medications per patient: 2.4, standard deviation: 3.0).

**Table 4:** Prevalence of, and attendance at secondary care for specific chronic illnesses same.

Chronic illness (ICPC code)	Number of patients with illness documented/number who have attended secondary care for this illness (cases)	
	'Cases'	'Controls'
Diabetes-Insulin Dependent (T90)	1/1	0/0
Diabetes – Non Insulin Dependent (T91)	1/1	1/0
Heart valve disease (K83)	1/1	0/0
Asthma (R96)	14/1	6/1
Chronic obstructive pulmonary disease (R95)	2/1	0/0
Depression (P76)	20/14	6/2
Viral hepatitis (D72): hepatitis C	38/24	1/0
Viral hepatitis (D72): hepatitis B	6/4	2/1
HIV/AIDS (B90)	8/8	1/1
Anxiety Disorder (P74)	2/1	1/1
Chronic alcohol abuse (P15)	5/0	0/0

Other chronic illnesses documented	47/27	52/27

Thirty-one patients (54%) had an acute illness during the previous three-months, (mean acute illnesses per patient: 1.0, standard deviation: 1.5). Upper respiratory tract infection (10 patients), sleep disturbance/anxiety/feeling depressed (four), abdominal pain (four), urinary tract infection (three) and ear wax (three) were the most commonly documented acute illnesses.

Twenty-five patients (44% of total) had been prescribed at least one time-limited medication (in addition to methadone) for the treatment of an acute illness during the previous three months (mean medications per patient: 0.9, standard deviation: 1.5).

### Health service utilisation among patients on methadone treatment

In the previous six months, patients on methadone had attended their GP for issues other than their addiction care a mean of 3.9 times (standard deviation: 4.1), and had attended another healthcare professional in the practice a mean of 0.5 times (standard deviation: 0.8). Three patients had used the out of hours services provided by the practice and ten patients (18%) had at least one diagnostic investigation arranged or performed by the practice. A total of 23 such investigations were performed on these ten patients, which included: biochemistry/haematology (13), microbiology (six), cervical smears (three) and radiology (one).

In the previous six months, 27 patients (47%) had either been referred to, or attended secondary care, with Emergency Department (10 referrals/attendances), infectious diseases (10 referrals/attendances), gastroenterology (8 referrals/attendances), hepatology (6 referrals/attendances), addiction services (4 referrals/attendances) and psychiatry (3 referrals/attendances) the secondary care services to which patients were most commonly referred/attended.

### Comparison with 'control' group

The mean age of 'controls' was 37.2 years (standard deviation: 0.44), 42(74%) were male, 41(72%) had GMS cover and all were Irish nationals. Thirty-eight (67%) were documented as living in stable accommodation.

Among the 'control' group (n = 57), morbidity and health service utilisation rates were also high, with 40(70%) having a documented chronic illness and 23(40%) being prescribed recurrent medications. In the previous six months, 27(47%) had consulted with a healthcare professional at the practice, 19(33%) were referred to secondary care and 11(19%) had investigations performed by the practice. In the previous three months, 21(37%) had attended with an acute illness.

Table 4 highlights a lower prevalence of psychological illness, bloodborne virus infection and respiratory illness among 'controls'. Table 5 compares morbidity and health service utilisation characteristics of patients on methadone with controls. Patients attending for methadone treatment were significantly more likely to have a chronic illness, to be prescribed recurrent medications and to consult with a GP or other healthcare professional at the practice. Although they were also more likely to have attended with and be prescribed medication for acute illnesses and to have been referred to secondary care, these differences were not statistically significant.

**Table 5:** Patients attending for methadone treatment compared to randomly sampled population matched by practice, age, gender and GMS cover.

	Patients on methadone	Randomly sampled control population	Odds ratio (95% confidence interval)	Chi squared (p value)
Chronic illness	52/57	40/57	4.4(1.5–13.0)	8.11(< 0.005)
On recurrent medications	39/57	23/57	3.2(1.5–6.9)	9.05(< 0.005)
Attendance with acute illness ^a^	31/57	21/57	2.0(1.0–4.3)	3.54(0.06)
Medication prescribed for acute illness ^a^	25/57	15/57	2.2(1.0–4.8)	3.85(0.05)
Attended GP/healthcare professional at practice^b^	45/57	27/57	4.2(1.8–9.5)	12.2(< 0.001)
Referred to/attended secondary care ^b^	27/57	19/57	1.8(0.8–3.8)	2.33(0.13)
Attended practices' out of hours/deputising service ^b^	3/57	5/57	0.6(0.1–2.5)	0.54(0.46)

Has had investigations performed/arranged by practice ^b^	10/57	11/57	0.9(0.3–2.3)	0.06(0.81)

^a ^in previous 3 months; ^b ^in the previous 6 months.

## Discussion

### Key findings

The proportions of cases (patients on methadone) and controls that had a chronic illness, that were prescribed regular medication, that attended with acute illnesses and that consulted with primary/secondary care were high, although patients on methadone treatment were more likely to have at least one chronic illness recorded, to be prescribed regular medications and to have attended primary care.

### Methodological considerations

Validity of the data reported in this paper is likely to have been enhanced by the practices in which it was conducted (research-active, with advanced practice information systems) and the method of data collection (two researchers validating and cross-checking data that had been extracted and a study instrument developed to minimise variation between researchers).

However, we acknowledge a number of potential sources of bias. Participating practices had a longstanding clinical and research interest in problem drug use. While not representative of practices providing methadone treatment in Dublin, we considered them an appropriate environment for exploratory research on this subject.

Ascertainment bias is also possible as patients on methadone must attend their GP every 1–2 weeks in Ireland [24]. As data were collected from clinical records, it is possible that frequent attendance (for methadone treatment) may increase documentation of chronic illnesses. It is also possible that this review of clinical records may have under/over reported other behaviours such as smoking. Selection bias was also possible, as patients with less severe and extreme problem drug use do not attend general practice for methadone treatment in Ireland.

Such considerations notwithstanding, the sample was comparable to larger samples of patients attending general practice for methadone treatment in Ireland in terms of gender and bloodborne virus status, but was older [27,28].

### How this relates to other literature

Work conducted in a similar setting to ours has identified a number of barriers to multimorbidity research and these include: problems with practice software, variations in disease coding and accurately determining primary and secondary care activity through clinical records [29]. While our experience would support these barriers and while morbidity data extracted from clinical records will inevitably be determined by clinical record keeping, we suggest that adopting a study instrument that is already being used in primary care [26] and modifying this to minimise inter-observer variation, can yield consistent data across practices.

Our findings regarding multimorbidity and chronic illness among patients on methadone treatment are consistent with North American studies that have reported higher prevalence of medical and psychiatric conditions among problem substance users [30,31]. Our findings regarding health service utilisation is consistent with other work which show illicit drug users more likely to use Emergency Department and primary care [32].

While chronic respiratory disease may not yet be widely recognised as such, this study highlights the potential importance of its prevention and treatment among problem drug users. We anticipated the prevalence of other chronic illnesses (eg chronic alcohol abuse, diabetes, hypertension, ischemic heart disease) might have been higher and reasons for this and incidence rates should be explored in further research, especially as the cohort ages. In time, it is possible that adverse lifestyle factors will increase the incidence of these chronic illnesses, thereby leading to more complex care.

This study has also documented a high prevalence of chronic illness, incidence of acute illness and high contact rates with primary/secondary care among 'controls', findings which may be explained by the study being conducted in areas of high deprivation [33].

### Implications for research and clinical practice

This pilot study has highlighted a need for further research on the epidemiology of chronic illnesses among patients on methadone. A larger, more representative sample of practices would make for more generalisable findings regarding illness prevalence. Conducting such research at practices from a range of socio-economic areas would allow controlling for deprivation as a potential confounder. Longitudinal studies would enable determination of incidence of key chronic illnesses, with data being collected directly from patients as well as from clinical records.

Such research should explore opportunities for primary and secondary prevention of chronic illness and determine uptake of primary care interventions in chronic illness management.

In this pilot study, a 21% difference in chronic illness prevalence rates between 'cases' and 'controls' was documented. However, chronic illness prevalence rates among problem drug users and 'controls' may have been higher in our study for reasons discussed above. Allowing for a more conservative estimate of chronic illness prevalence among (general) young adult populations of 48% [34], a 21% difference among problem drug users, we estimate data on 86 patients on methadone and 86 controls would determine a statistically significant difference in chronic illness prevalence rates (assuming significance of 0.05, power of 0.90 and a case:control ratio of 1:1).

If the findings of this pilot study are supported by more powerful/representative studies, then screening and treatment of chronic illness and increasing care complexity will be important issues in the future management of problem drug users. Therefore, an integrated care model, in which primary care and addiction care both care for problem drug users may best address this population's health needs [35].

## Conclusion

Multimorbidity is common among problem drug users attending general practice for methadone treatment, and primary care may have an important role in primary and secondary prevention of chronic illnesses among this group. Further work on chronic illness and health service utilisation among problem drug users is advocated and this study offers a feasible study instrument.

## Competing interests

The authors declare that they have no competing interests.

## Authors' contributions

WC conceived the study, WC, GB, AOC, FDOK wrote the proposal, SOB, WC, AOC, FDOK collected the data, WC, SOB analysed the data. WC drafted the initial manuscript. WC drafted subsequent manuscripts in response to editorial comments. All authors read and approved the final manuscript.

## Appendix 1. Final draft of study instrument

### A. Administrative details

HSE area of practice

East Coast

Northern

South western

Southern

NW

Midland

Western

Mid western

NE

Type of addiction treatment

Being prescribed methadone at present:   Yes         No

Primary agency managing addiction problem: GP         Addiction clinic

Client number ____________(Note deleted from final electronic file)

### B. Demography

Gender               Male      Female

Age last birthday

DOB

Health cover            GMS      Non-GMS

GMS number (Note deleted from final electronic file)

Living where

Stable accommodation

Institution

Homeless

Other

Not Known

Area of residence

DED            ______________

CCA            ______________

County registration plate      ______________

ationality

Irish

Irish traveller

Other

Not known

### C. D. E. Addiction care details

Date first referred to/attended general practice   _____________

Main reason for first referral/attendance (tick)

Alcohol use

Illicit drug use

Licit drug use

Referred by specialist addiction services for methadone treatment

General medical care/GMS registration

Other

Was the patient prescribed methadone by any other agency/clinic before attending the practice for methadone treatment:

                     Yes      No

What is the earliest recorded date on which the patient was prescribed methadone by any agency/clinic (01/month/year)         _____________

What is the earliest recorded date on which the patient was prescribed methadone at this practice (01/month/year)            _____________

Has the patient attended any other agency/clinic for addiction treatment since the above date (21b)?

                     Yes      No

Has the patient attended any other agency/clinic for addiction treatment in the last 12 months?

                     Yes      No

Date last treatment episode with methadone commenced _____________

Dose of methadone at last prescription _________MILLIGRAMMES PER DAY

### F. Chronic disease

Which illnesses that require ongoing follow up have been documented at the time of data being collected and/or at any time in the past and in the case of each has patient attended secondary care for this problem

- Diabetes-Insulin Dependent (T90)

- Diabetes – Non Insulin Dependent (T91)

- Cardiac arrhythmia (K80)

- Rheumatic fever (K71)

- Ischaemic heart disease with angina (K74)

- Acute myocardial infarction (K75)

- Ischaemic heart disease (K76)

- Heart failure (K77)

- Pulmonary heart disease (K82)

- Heart valve disease (K83)

- Heart disease other (K84)

- Hypertension complicated (K87)

- Asthma (R96)

- Chronic obstructive pulmonary disease (R95)

- Depression (P76)

- Viral hepatitis (D72): hepatitis C

- Viral hepatitis (D72): hepatitis B

- HIV/AIDS (B90)

- Anxiety Disorder (P74)

- Chronic alcohol abuse (P15)

- Other chronic illnesses

Documented acute illnesses in the preceding 3 months (and their ICPC-2 code)

If the patient attended today for a repeat prescription, what medication would be prescribed? Medication (generic name)/route

Acute/non-recurrent medications in the last 3 months: medication (generic name)/route

In the last 6 months, number of consultations (excluding those concerning ONLY methadone) with:

- a doctor in the practice:            ____________

- another healthcare professional in the practice:   ____________

Has patient been referred to or attended secondary care (including emergency departments) in the last 6 months?

                     Yes      No

If yes, please specify: specialty/date referred by practice/date attended specialty

Has patient attended out of hours/GP deputising service in the last 6 months?

                     Yes      No

If yes, please specify: Date/Problem/ICPC code

Has patient had a diagnostic investigation arranged/performed by the practice in the last 6 months (excluding urine toxicology)?      Yes      No

If yes, please specify: pathology/diagnostic imaging/other

## Pre-publication history

The pre-publication history for this paper can be accessed here:


